# Original Communication

**Published:** 1841-02-20

**Authors:** Henry Wheaton Rivers


### ORIGINAL COMMUNICATION.

*Providence, Feb.* 8, 1841.

To the Editors of the Medical Examiner.

*Gentlemen*,—I have this day received the following letter from *Samuel Y. Atwell, Esq.*, of this city, in which he gives the credit of having first suggested the operation for strabismus to *Dr. Wm. Ingalls*, of Boston.

*Mr. Atwell* is an eminent member of the legal profession in this state, and his statements are worthy of the highest credit.

I think it due to *Dr. Ingalls* that the fact of his having first suggested the operation, should be made known to the profession.

I also send you the notes of two cases of strabismus on which I have operated successfully.

Your obedient servant,
Henry Wheaton Rivers, M. D.

*Providence, Feb.* 8, 1841.

*Dear Sir*,—I observe from the newspapers that you have operated with great success in several cases of strabismus, or squinting. I have also noticed that this operation is spoken of as a new discovery in the art of surgery, and is said to have lately originated in Germany. Now, sir, I think we should give honour where honour is due. In the years 1812 and '13, I attended courses of surgical and anatomical lectures delivered before the Medical School of Brown University, by *William Ingalls*, M. D., of Boston, then the Professor of Anatomy and Surgery in that institution: being subject myself to this infirmity, (strabismus,) Dr. Ingalls took frequent opportunities to explain to me the method of its surgical cure; he did this by dissection of the eye itself, explanation of the power and disposition of the several muscles appertaining to that organ, and showed me how, by a division of one or more of them, the eye might be brought to its proper place. In my own case I know he proposed to divide the *rectus internus*. So strongly was I impressed with the practicability and success of this operation, that I strongly urged my father to permit me to submit to the operation; but upon the nature of the operation being explained to him, he declined the permission, because he feared the effect might be to turn the eye the other way.

I make this statement in justice to my friend and quondam master, and to show that we have some surgeons in this country as learned in their profession as some in Europe.

Respectfully your obedient servant,

SAMUEL Y. ATWELL.

*To Henry W. Rivers, M. D., Providence, R. I.*

*Case* I.

STRABISMUS DIVERGENS—*Division of the Rectus Externus.*

*Dec.* 23, 1840. Elizabeth N., æt. nineteen, has had an outward turn in her right eye from birth. At her request, I performed the operation of dividing the external rectus muscle this day. The eye immediately became straight. She was directed to keep the eye covered, and constantly wet with cold water; her diet to be light for a few days.

24th. Has had no pain in the eye; ecchymosis considerable; applied two foreign leeches to temple, and ordered a dose of magnes. sulph.

27th. Ecchymosis less; no pain in the eye; continue to bathe it with cold water. A slight fungus projects from the wound, which is touched daily with the argent. nit.

Jan. 11th, 1841. Eye well, and perfectly straight.

*Case* II.

STRABISMUS CONVERGENS—*Division of the Rectus Internus.*

J. H. M., æt. thirty-eight, has had strabismus convergens of his right eye since his fourth year, it being the sequel of pertussis. The vision of this eye is so imperfect that he can only distinguish that there is a dark object in the way, when a person stands before him at the distance of four or five feet.

*Operation January* 13*th*, 1841.—The eye became straight immediately. The eye was covered with a compress wet with cold water, and confined by a light bandage. Cold to be applied through the day.

*Evening.* Can distinguish a person's features at the distance of four or five feet. Continue cold.

14th. Vision improving; can distinguish some apples on the head of a barrel at the distance of five or six feet; eye perfectly straight; ecchymosis extends from semilunar fold to within a line or two of cornea.

16th. Ecchymosis about the same; a slight fungus appears at the wound, which is touched with the argent. nit., which is to be repeated daily. Directed to cover sound eye, and exercise the other two or three hours at a time.

18th. Eye continues straight; redness diminishing.

Jan. 31st. The eye is well; perfectly straight, and with the exception of a little redness, which is daily diminishing, looks perfectly natural.

I have within a few days operated on three other cases of strabismus convergens, with every prospect of success.

H. W. RIVERS, M. D.

